# Subtyping of Adolescents with School Refusal Behavior: Exploring Differences Across Profiles in Self-Concept

**DOI:** 10.3390/ijerph16234780

**Published:** 2019-11-28

**Authors:** Carolina Gonzálvez, Ángela Díaz-Herrero, María Vicent, Ricardo Sanmartín, Antonio M. Pérez-Sánchez, José M. García-Fernández

**Affiliations:** 1 Department of Development Psychology and Teaching, University of Alicante, 03690 San Vicente del Raspeig, Spain; carolina.gonzalvez@ua.es (C.G.); maria.vicent@ua.es (M.V.); ricardo.sanmartin@ua.es (R.S.); am.perez@ua.es (A.M.P.-S.); josemagf@ua.es (J.M.G.-F.); 2 Department of Development Psychology and Education University of Murcia, 30003 Murcia, Spain

**Keywords:** school refusal behavior, self-concept, latent class analysis, adolescence

## Abstract

Not all adolescents with school attendance problems attribute their behavior to the same causes. Knowing the subtypes of students who reject school and their relationship with new variables, such as self-concept, is an unresolved task. This study aimed to identify different school refusal behavior profiles and to determine whether these profiles differed from each other based on the scores of the eleven dimensions of self-concept (Physical appearance, Physical abilities, Parent relations, Same-sex relations, Opposite-sex relations, Honesty, Emotional stability, Self-esteem, Verbal, Math, and General school). The participants were 1315 Spanish students (57.6% male) aged 12–18 years (M = 15.21; SD = 1.74). The School Refusal Assessment Scale-Revised and the Self-Description Questionnaire II-Short Form were administered. A latent class analysis revealed four school refusal behavior profiles: Moderately High School Refusal Behavior, Moderately Low School Refusal Behavior, Mixed School Refusal Behavior and Non-School Refusal Behavior. The results indicated that the Mixed School Refusal Behavior group was the most maladaptive profile and revealed the lowest mean scores on self-concept. In contrast, Non-School Refusal and Moderately Low School Refusal Behavior groups revealed the highest scores in all dimensions of self-concept. Implications for working toward the prevention of school refusal in students with low self-concept are discussed.

## 1. Introduction

School attendance problems represent an important social and public health problem due to their negative consequences on the development of children and adolescents [1]. It is common for young people with school refusal behavior (SRB) not to finish compulsory education and drop out of school, which is associated with economic, medical, psychosocial, and marital problems in adulthood [2,3,4,5,6]. In addition, SRB is a risk factor for other negative behaviors that are manifested especially in adolescence, such as poor academic performance, teenage pregnancy, violence, poor psychological well-being, substance use, and criminal behaviors [7,8,9,10]. This empirical evidence highlights the importance of an early detection of the factors or variables associated with SRB and this study was framed in this line. However, this work is hindered, to a large extent, by the diversity of perspectives regarding the conceptualization, classification and etiology of school attendance problems [11].

Despite these difficulties, Heyne et al. [11] argued that we can distinguish two major contemporary approaches in the study of school attendance problems. One of them, supported by Heyne and colleagues [11], supports the differentiation between types of school attendance problems, distinguishing between school refusal, truancy, school withdrawal and school exclusion. These authors, for the evaluation of these typologies, developed the School Non-Attendance ChecKlist (SNACK) [11]. The other approach undertaken in this research was based on the functional analysis model developed by Kearney and Silverman [12]. The four functional conditions proposed by this model were assessed through the School Refusal Assessment Scale-Revised (SRAS-R) [13]. This instrument has been widely used outside the United States, where it was initially developed, and has demonstrated adequate psychometric properties in populations of both European and Asian countries [14,15,16,17,18,19,20,21] as well as Latin Americans [22,23].

Based on this model, four functional conditions or factors were established as motivators of SRB: (1) Avoiding the negative affectivity caused by school-related stimuli; (2) escaping from social situations and/or aversive evaluation; (3) seeking the attention of other significant people; and (4) seeking tangible reinforcements outside the school environment. The first two conditions of SRB are maintained by negative reinforcement, which implies that although they are not receiving any reward, they are being excused from a situation or environment that they find unpleasant or aversive. Regarding the third and the fourth functional conditions, adolescents’ SRB is maintained by positive reinforcement, that is, by refusing to attend school so that they can remain with a significant person (e.g., parents) or be doing activities that they consider more pleasant (e.g., playing video games).

### 1.1. School Refusal Behavior Profiles

According to Kearney [24], SRB can be caused by several causes or reasons at the same time. On that basis, some authors have tried to establish profiles or categories of people with SRB using mainly factor analysis [25,26,27,28,29,30,31,32,33]. Establishing groups with specific characteristics would facilitate being able to carry out more effective interventions in line with the needs of each profile.

In this line, we can highlight the study carried out by Dube and Orpinas [27] and Gonzálvez et al. [29,30,31,34] from the approach of the functional model proposed by Kearney and Silverman [12]. Specifically, Dube and Orpinas [27] identified three profiles: A mixed or multiple SRB profile, since it included explanatory factors for both positive and negative reinforcement (high scores in the first, the second and the fourth factor of the SRAS-R), an SRB profile by positive reinforcement (high scores in the third and the fourth factor) and a non-SRB profile. These results were obtained with a non-clinical sample composed of 99 American students aged between 8 and 15 years (M = 12.5; SD = 1.38).

From this same perspective, Gonzálvez et al. [29,30,31,34] have recently carried out various works with community samples that regularly attended school. In these works, various SRB profiles with large samples of children and adolescents were detected by cluster analysis. More specifically, Gonzálvez et al. [29,30,34] revealed four SRB profiles of Spanish children (*n* = between 1113 and 1212 participants) aged between 8 and 11 years. Three of them were similar in these studies and were named Non-school refusers, School refusers by mixed reinforcements and School refusers by negative reinforcements. However, the fourth profile was called School refusers in two of those studies by positive reinforcements (high scores in the third and the fourth of the SRAS-R) [29,30], and School refusers by tangible reinforcements (high scores in the fourth of the SRAS-R) whereas, in the other study, they did not match exactly [34]. More recently, a latent class analysis study, which is a more sophisticated statistical technic to detect profiles, revealed four SRB profiles with some of them previously unidentified: Moderately Low SRB, Moderately High SRB, High SRB, and Non SRB [35]. The groups with the word “moderately” in their names had not been identified before. However, the individuals contained within them did not reach sufficiently high or low scores and the tendency on their scores was either in positive or negative orientation. In addition, a group with high scores in the four factors of the SRAS-R was identified, the High SRB profile. These results were obtained with a non-clinical sample composed of 1842 Spanish adolescents aged between 15 and 18 years (M = 16.43; SD = 1.05).

In addition, in another study with 1582 Ecuadorian adolescents aged between 12 and 18 years old (M = 14.83; SD = 1.86), the cluster analysis revealed three SRB profiles already identified above. These profiles were Non-school refusers, School refusers by mixed reinforcements, and School refusers by tangible reinforcements [31]. All these studies agreed that students who were part of the group School refusers by mixed reinforcement showed the most psychological and social adjustment problems since they obtained lower scores in social functioning and higher scores in anxiety, depression and stress [29,30,31,34].

### 1.2. School Refusal Behavior and Self-Concept

School attendance problems have been associated with various personal variables that affect the adjustment and well-being of children and adolescents. Specifically, SRB has been associated with low self-esteem [36,37], internalizing and externalizing symptoms [38], bullying [39], academic difficulties [33], and thoughts of personal failure [40]. However, another individual perception that could be influencing SRB is self-concept. As Bandura [41] pointed out, thoughts and feelings about oneself are related to people’s ways of acting, especially when faced with challenging situations. In the educational context, students must be able to face numerous challenges, such as relating competently with their classmates, passing academic evaluation tests, and showing motivation for learning, which do have an impact on their academic adjustment.

Traditionally, the study of self-concept has given rise to two main theoretical currents that have defended either the unidimensional or the multidimensionality of the construct. However, today, it advocates a hierarchical and multidimensional conception of self-concept [42,43]. From this perspective, it is considered that this construct refers to the set of perceptions that form the image that a person has of themself and in its configuration, both cognitive and social aspects come into play [43,44]. Thus, it is especially influenced by the evaluations of other significant people, by the reinforcements of the environment and by the attributions about the behavior itself [42]. In addition, in terms of its structure and hierarchical organization, it is argued that the general self-concept distinguishes, on a first level, between academic and non-academic self-concept, dividing these components, in turn, into different more specific subcomponents [42,43].

In adolescence, the construction of the self-concept occupies a central place, being considered one of its main challenges [45]. The numerous biophysiological, cognitive, social, emotional, and affective changes that occur at this stage of development influence the formation of self-concept, and promote that young people carry out a review of their self-image [46,47].

In addition, the study of self-concept in adolescence is of vital importance since it is considered an important indicator of psychological well-being and personal, social, and school adjustment [48,49]. In this line, studies that have been carried out under a multidimensional perspective have found that adolescents who are involved in aggressive behaviors have a lower family, academic and social self-concept than their peers who do not show this type of behavior [50,51]. Similarly, other authors observed negative and statistically significant correlations between state anxiety and trait anxiety and academic, social, and family self-concept [52]. Moreover, the observed relationships between self-concept and social and school anxiety point in the same direction [53,54,55]. As an example, Delgado et al. [53] found that adolescents with social anxiety were more likely to negatively perceive their relationships with partners of the opposite sexes and to have low self-esteem. In addition, in the study by Gonzálvez et al. [55], statistical analyses revealed that students with low scores in most dimensions of self-concept showed significantly higher scores in school anxiety than their peers with high scores in self-concept.

However, the relationships between self-concept and school refusal behaviors have hardly been analyzed in the scientific literature. In fact, we can only review a few empirical evidences of the links between the two constructs. On the one hand, Kearney and Silverman [56], in a study carried out with 42 North American children with an average age of 11 years, to analyze the psychometric properties of the School Refusal Assessment Scale (SRAS), found that the self-concept evaluated by the Piers-Harris Self-Concept Scale (PHSCS) [57] showed negative and significant correlations with the first two functional conditions of SRAS (*r* = −0.31, *p* < 0.05; *r* = −0.46, *p* < 0.01, respectively). On the other hand, Reid [36] investigated the relationship between academic self-concept and self-esteem with 308 English children from a deprived area in South Wales (United Kingdom) comparing a group with persistent school absenteeism and two control groups who regularly attend to school. The results showed that absentees had significantly lower self-concept and self-esteem in comparison with their peers’ regular attenders. According to Ahmadi, Najafi and Khanehkeshi [58], those adolescents who do not regulate or have a lack of self-efficacious behaviors have lower school motivation and consequently, failure at schools, poor academic performance, maladaptive behaviors at home and even school absenteeism or drop out could affect them. In this sense, scientific findings in a sample of 120 students between 15–16 years old revealed that school refusal behavior students obtained low scores than normal groups in academic self-efficacy and self-regulation [58].

Balkis, Arslan, and Duru [59], in a study carried out with 423 Turkish students aged between 15 and 18, found negative and significant correlations between academic self-perceptions and school absenteeism. As in other previous studies [37,60], it was confirmed that students with high rates of school absenteeism had a negative view about their general and academic competence. However, these statements cannot be considered totally conclusive since in the research of Hassan, Jami, and Aqeel [61], although the students who regularly attended class, compared to the truant students, showed higher scores in academic self-concept, the differences did not reach statistical significance. This study was conducted with a sample of 200 Pakistani adolescents (truant = 100, punctual = 100) between 12 and 18 years of age. On the basis of these findings, more research in this field is required to determine whether self-concept is a decisive factor in school adjustment and school attendance problems [62].

### 1.3. The Present Study

Previous research analyzing the relationship between SRB and self-concept is very scarce and suffers from numerous limitations at the methodological level. First, in these studies, SRB have not been framed in any theoretical model, with the exception of the study by Kearney and Silverman [56], which makes it difficult to compare their results. The few investigations carried out in this regard [59,61] have simply focused on school absenteeism and have analyzed their relationships with students’ academic self-perceptions. For the study of school attendance problems, the functional model of Kearney and Silverman [12] represents a solid and adequate theoretical framework that argues that SRB can be caused by various causes or reasons at the same time, including both behaviors of school refusal related to anxiety when faced with school situations, such as those more closely related to truancy.

Secondly, no previous study has been found that has sought to assess the relationships between school attendance problems and the dimensions of self-concept using the short version of the Self-Description Questionnaire II-Short Form (SDQ-II-S). This questionnaire represents one of the most frequently used multidimensional measures of self-concept, given its adequate psychometric properties and construct validity [52,63,64]. On the other hand, as we have already pointed out, previous studies have only focused on academic self-concept, without taking into account the other dimensions or facets of self-concept. It should be noted that the multidimensional conception of the self-concept on which this research is based is widely accepted by the scientific community [43,48] and allows us a more specific analysis of the relationships between this construct and the problems of school attendance.

Thirdly, the results of previous research have been analyzed using statistical techniques such as correlational analysis or Student’s t-tests. In this sense, studies using a more rigorous statistical methodology that allows us to reach solid empirical evidence are required.

Given these limitations, the present study sets out two main aims. The first aim was to identify, through latent class analysis (LCA), SRB profiles in a large community sample of Spanish adolescents based on the four explanatory factors of the SRB assessed using the SRAS-R. The second aim, once the profiles were established, was to analyze whether there are statistically significant differences in the scores of the dimensions of the self-concept belonging to the different profiles.

Although there is no previous research that specifically tried to analyze the role of self-concept in SRB from this perspective, some authors have highlighted relationships between self-concept and anxiety [29,30,53,55] and between school absenteeism and academic self-concept [59,61]. Based on these considerations, the following hypotheses were proposed: (1) That four different profiles of students with SRB would be identified based on the functional model of Kearney and Silverman [56] and on the findings of Gonzálvez et al. [29,30]. Specifically, it was expected to identify four profiles: Non-school refusers, School refusers by mixed reinforcements, School refusers by negative reinforcements and School refusers by positive reinforcements; (2) that students’ groups with school refusal behaviors’ problems will be associated with worse scores in self-concept [36,56,59,60].

## 2. Materials and Methods

### 2.1. Participants

A random cluster sampling technique was used to collect the sampling unit at the high schools. Of all education centers of Secondary Compulsory Education in the province Alicante (geographical area located at the southeast of Spain), eleven high schools were randomly selected. Eligible individuals were 12–18 years of age (*M*_age_ = 15.21; *SD* = 1.74). The initial sample was composed of a total of 1413 students. Of these, 62 (4.4%) were excluded due to coding errors during the tests, 31 (2.1%) were excluded for not having the written consent of their legal tutors to participate in the research and 5 (0.4%) did not have good enough level of Spanish to understand the items, resulting in a total of 1315 students (57.6% male). Regarding the sociocultural and economic context, based on the educational level and jobs of the families, the middle-class families predominated.

### 2.2. Procedure

Prior to administering the tests, a meeting was carried out with the principals of each high school to explain the aim of the study and informed consent was then sought from the students’ parents or legal guardians. Regarding data collection, the instruments were administered at the high schools themselves during normal class periods (approximately 30 min). All sessions were supervised by a member of the research team who had previously received instruction in the procedures. The voluntary nature of participation and the need for sincerity were emphasized. The Ethics Committee of the University of Alicante (File number: 20170905) approved the study and the standards established by the Declaration of Helsinki (1964) were followed.

### 2.3. Measures

School refusal behavior was assessed using the School Refusal Assessment Scale-Revised (SRAS-R) [13]. The SRAS-R is a self-report measure with 24 items that assess four functional conditions for the maintenance of school refusal behavior: (1) Avoidance of school-related stimuli that provoke a sense of general negative affectivity, (2) escape from aversive social and/or evaluative situations at school, (3) pursuit of attention from significant others, and (4) pursuit of tangible reinforcement outside of the school setting. In this study, the Spanish version consisting of 18 items divided into the same four factors and whose levels of reliability ranged from 0.70 (Factor I) to 0.87 (Factor III), was used [16]. Each item was scored on a seven-point Likert scale (0 = never; 6 = always). The coefficients of internal consistency (Cronbach’s alpha) obtained in the present study ranged from 0.71 (Factor IV) to 0.84 (Factor II).

Self-concept was assessed using the Self-Description Questionnaire (SDQ–II-Short Form) [63]. The SDQ-II-Short is a self-report measure to assess self-concept in adolescents aged between 12 and 18 years old. This questionnaire is formed by 51 items scored on a 6-point Likert scale (1 = false; 6 = true), in comparison with the original version proposed by Marsh [65] consisting of 102 items. The SDQ-II-Short Form assesses adolescents’ self-concept loaded into eleven scales: Three academic dimensions (Verbal, Math, and General school), seven non-academic dimensions (Physical appearance, Physical abilities, Parent relations, Same-sex relations, Opposite-sex relations, Honesty, and Emotional stability) and a dimension about self-esteem. Subsequent studies have replicated the eleven factors of the instrument and support its reliability [64,66]. The coefficients of internal consistency (Cronbach’s alpha) obtained in the present study ranged from 0.60 (Opposite-sex relations) to 0.77 (Physical appearance).

This instrument has been adapted for the Chilean population, corroborating its factor structure and showing adequate reliability and validity indices [51]. In this study, the Latin American version of the scale was used and the coefficients of internal consistency (Cronbach’s alpha) obtained ranged from 0.70 (Opposite-sex relations) to 0.80 (Math).

### 2.4. Data Analysis

First, latent class analysis (LCA) was performed to classify participants based on school refusal behavior scores across the four functional dimensions of the SRAS-R. LCA is considered a more precise technique that overcomes the limitations presented related to K-means clustering [67]. The Bayesian information criterion (BIC) and the Entropy values were used to assess the model fit to determine a suitable number of latent classes. The lowest values for the BIC and closed to one for Entropy were considered the fit indices taken into account in order to choose the most adequate class solution [68]. In addition, it was considered the theoretical feasibility and psychological significance of each of the groups that represents the different school refusal behavior profiles and maximizes the inter-classes differences.

Once the school refusal behavior profiles were determined, the inter-class differences in the obtained scores on the seven dimensions of self-concept were analyzed using analysis of variance (ANOVA). In addition, a Bonferroni method was carried out to determine the post hoc tests and effect sizes (Cohen’s d index) were calculated for the observed differences. Specifically, d levels between 0.20 and 0.49 indicate a small effect magnitude, between 0.50 and 0.79 indicate a moderate magnitude, and a value equal to or greater than 0.80, a large one [69].

## 3. Results

### 3.1. School Refusal Behavior Profiles

**Figure 1:**
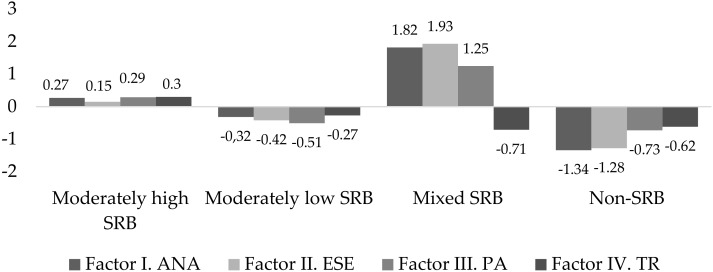
School refusal behavior profiles. SRB: school refusal behavior.

Four consecutive latent class models were estimated to identify the underlying class structure within the sample. Table 1 shows the BIC and entropy scores for the four latent class models. The four classes reached the highest entropy scores, whereas the lowest BIC value was obtained by the five classes followed very closely by the four classes. In addition to these statistical criteria, in order to maximize the inter-classes differences and considering the theoretical meaning of the classes obtained the four-class model was identified as the best fitting model. The first class, Moderately High School Refusal Behavior included 489 students (37.2%) characterized by moderate levels in all SRAS-R dimensions. The second class, Moderately Low School Refusal Behavior, included 433 students (32.9%) having low levels of school refusal behavior by pursuit attention from significant others and moderately low in the rest of SRAS-R dimensions. The third class, Mixed School Refusal Behavior, classified 177 students (13.5%) with high levels of school refusal behavior in the first three factors and low in the fourth factor of the SRAS-R; whereas the last class, Non-School Refusal Behavior, included 216 students (16.4%) with low scores of school refusal behavior in all dimensions (see Figure 1).

### 3.2. Inter-Class Differences in Self-Concept

Table 2 presents the means and standard deviation for the eleven dimensions of self-concept across the four classes of school refusal behavior identified. Statistically significant differences were revealed in all cases. The Mixed SRB group obtained the lowest means in all dimensions of self-concept, except for the verbal self-concept, since the Moderately High SRB class was the group that obtained the lowest average score. In contrast, the Non-SRB class attained the highest means all dimensions of self-concept, except for the three dimensions related with academic issues (Verbal, Mathematics and Academic General self-concept) since the Moderately Low SRB class was the group that obtained the highest average scores in these three dimensions.

The post hoc comparisons revealed that the effect size magnitude of these differences ranged from 0.16 to 0.98 (see Table 3). The largest effect sizes were found between the Mixed SRB and the Non SRB with a large effect size for the Emotional stability, Opposite-sex relationships, and Same-sex relationships dimension, moderate magnitude for the Physical appearance, Self-esteem, Honesty, and Parent relations dimensions, and, finally, small effect size for Physical abilities, Math, and General school dimensions. Similar results were found between the Mixed SRB and the Moderately Low SRB groups. In contrast, not statistically significant differences were found between the Non-SRB and the Moderately Low SRB.

## 4. Discussion

The relationships between SRB and self-concept have barely been studied in the scientific literature. The few existing studies have focused mainly on a single typology for school attendance problems, school absenteeism or truancy, relating it to academic self-concept [59,61]. In fact, to date, there have been no investigations with large community samples that take into account the causal heterogeneity of SRB and the multidimensional conception of self-concept.

The present study sought to solve this emptiness by providing new empirical evidence on the relationship between these two constructs, using a rigorous statistical methodology. Our purpose was reflected in two aims: To identify, from the functional model of Kearney and Silverman [56], the SRB profiles in a large community adolescent sample and analyze their relationships with self-concept, taking into account its three academic scales (Mathematical, Verbal, and General academic), seven non-academic (Physical appearance, Physical skills, Relationship with parents, Same sex relationships, Relationships with the opposite sex, Honesty, and Emotional stability), and the scale of Self-esteem.

Through latent class analysis, four profiles of students with SRB were distinguished (Moderately High School Refusal Behavior, Moderately Low School Refusal Behavior, Mixed School Refusal Behavior and Non-School Refusal Behavior). Two of these groups, Mixed School Refusal Behavior and Non-School Refusal Behavior, coincided with those established in the first hypothesis. These profiles showed similarity with those identified in previous studies [27,29,30,31,34] and were characterized, in the first case, by high scores in the first three factors and low in the fourth factor of the SRAS-R and, in the second, by low scores in all the factors of the SRAS-R.

In contrast, the Moderately High School Refusal Behavior and Moderately Low School Refusal Behavior profiles revealed two groups with a high and low tendency levels of SRB, respectively, in all dimensions of the SRAS-R but that in none of the cases significant scores are reached to be considered high or low scores. These profiles coincide with the groups identified by a recent research also carried out with Spanish adolescents [35]. The configuration of these new profiles suggests that the four functional conditions of the SRAS-R are not mutually exclusive and that there are relationships between them. This fact has been shown in some studies that, based on the functional model, have detected a mixed profile which includes high scores in factors for both positive and negative reinforcement [27,29,30,31,34]. In addition, other studies that have taken into account the classification of different types of school attendance problems, have found correlations between school refusal and truancy [39]. Therefore, these results only allowed us to partially confirm the first hypothesis and highlight the need for further research with LCA to corroborate these profiles.

Regarding the relationships between SRB and self-concept, the data confirmed the second hypothesis raised in the study which established that students’ groups with school refusal behaviors’ problems would have worse scores in self-concept. According to this idea, the Mixed School Refusal Behavior was characterized by the highest SRB scores and obtained the lowest scores in all the dimensions of self-concept, except in the verbal self-concept in which the lowest average score was obtained by the Moderately High SRB group, although no significant differences were found between them. This fact identifies the Mixed School Refusal Behavior as the most maladaptive profile in terms of average self-concept scores, followed by the group with Moderately High SRB. These results are consistent because these two groups are the only profiles characterized by high orientation scores in SRB. More specifically, adolescents belonging to the Mixed School Refusal Behavior group showed the lowest scores in physical self-concept, social self-concept, general and mathematical academic self-concept, honesty, emotional stability, and self-esteem, compared to the other profiles. These results could be justified by the statistically significant and positive correlations found among the three factors of the SRAS-R in which these students reach high scores and different anxiety disorders and depression [70]. Previous studies have warned of the maladaptive nature of this profile, being the group that has reported the worst results in social anxiety [35], social functioning [34], or anxiety, depression, and stress [31].

On the contrary, the Non-School Refusal Behavior group was the one that showed the highest average scores for all the dimensions of self-concept, except for the three academic dimensions (Verbal, Mathematical, General Academic) whose highest scores were obtained by the Moderately Low SRB profile, although again, these differences were not statistically significant. Even though no significant differences in the effect sizes were found between the Non-School Refusal Behavior and Moderately Low School Refusal Behavior profiles, the scores reported by these classes in the three academic self-concept dimensions require further reflection. These results may be explained by the intensity scores in the four factors assessed by the SRAS-R. From the four factors that SRAS-R evaluates, the second factor refers to the need to escape from situations of social aversion and evaluation. In this factor, assessment is also associated with academic aspects and based on these results, it seems that obtaining non-significantly low scores in this concern makes them scoring better in those academic dimensions of self-concept. However, the interpretation of these results must be cautious since the differences between the two groups do not reach a significant effect size so it is necessary to expand the investigations in this regard.

In this study, young people associated with the Mixed SRB profile showed more negative self-perceptions regarding their competence in the academic field and in terms of their social relationships with both their parents and peers of the same and different sex, are considered less attractive or athletic on the physical level, show lower self-perceptions of emotional well-being and have greater feelings of dissatisfaction with respect to themselves. These results are in line with those obtained by Kearney and Silverman [56] where it was observed that the self-concept showed negative and significant correlations with the first two functional conditions of SRAS. These findings suggest that SRAS-R factors that are related to anxiety or discomfort in school situations are those that show a greater association with self-concept, consistent with our findings. However, these results could contradict those of the study by Balkis et al. [59] that indicated negative and significant correlations between school absenteeism and academic self-concept, although this association have not been confirmed in other investigations [61].

These findings demonstrate the negative relationship between the Mixed School Refusal Behavior profile and various dimensions or facets of self-concept. Together with other personal factors, self-concept was revealed as an important affective-motivational variable in the explanation of school attendance problems. Therefore, having a good self-concept could be, as our data suggests, a factor of protection against school attendance problems. In fact, Balakrishnan and Andi [71] emphasize the importance for parents and teachers of talking to school refusers and giving them academic motivation and increasing their self-concept and self-efficacy. As Reid [36] points out it is possible that students who constantly obtain low grades in school can perceive their academic self-concepts reduced to a level that to missing school becomes a source of relief. However, the SRB prevention should not only consider the student’s personal factors because there are other school factors, such as the relationship with peers or the teachers’ classroom management, which could be potentially risk factors for SRB [72].

Finally, this study presents some limitations that should be highlighted. First, comparisons with other investigations were not possible since the few existing studies on the subject focused on analyzing the relationships between school absenteeism and academic self-concept (e.g., 59,61). Therefore, future research should analyze the relationships between SRB and self-concept to verify whether these results coincided with those obtained in samples of other age ranges, different sociocultural and economic contexts or using other multidimensional self-concept measures (e.g., AF5 Self-Concept Questionnaire) [73]. In addition, other psychological variables such as academic self-efficacy, aggressiveness, or even social background should be considered in future studies to continue working on this topic. Secondly, given the cross-sectional nature of the study, it was not possible to draw conclusions regarding the causal relationships between the analyzed variables. Therefore, prospective longitudinal studies and the use of structural equation models are advisable. Thirdly, in this research, only student self-report measures were used. It would be convenient for future studies to adopt a multi-method and multi-source evaluation perspective using not only self-report measures, but also interviews, observational measures, assessment scales, checklists, school attendance records, and information collected from others. Key informants such as parents, teachers and classmates should be considered. Finally, it would be advisable to complement these findings with the approach of Heyne et al. [11], based on the differentiation between school attendance problems. This could give us a broader view of the differences between young people with school attendance problems.

Despite these limitations, the results of this study are very relevant because they offer, for the first time, an exhaustive analysis of adolescents’ self-perceptions with school attendance problems. These findings highlight the need to carry out prevention programs focused on fostering interpersonal relationships and reducing the emotional distress that students may be experiencing in different school situations, as well as designing policies according to the profiles’ characteristics. During adolescence, self-concept is crystallizing, while it is going through a period of revision and refinement. Taking advantage of these circumstances, education professionals should promote a positive self-concept in students by providing them with an organized and supportive learning environment in which they are adjusted and positive perceptions of themselves in different fields, appropriate learning strategies and self-control, social and emotional skills, and healthy attributional styles. However, for all these actions aimed at reducing school attendance problems to be successful, a broad and close collaboration between parents and professionals in the educational field is necessary [24,27].

## 5. Conclusions

This study contributes by verifying the existence of different profiles of students with SRB and their relationship with self-concept. The study explicitly differentiated between four SRB groups: Non-School Refusal Behavior, Mixed School Refusal Behavior, Moderately Low School Refusal Behavior, and Moderately High School Refusal Behavior. The Mixed School Refusal Behavior profile was the group with the most maladaptive scores in self-concept. As in previous studies, this group reported worse scores in personal and internalizing problems (e.g., anxiety, depression or stress) warning of the problems associated with this profile [29,30,31,34]. This implies that groups with high scores of school refusal behavior for positive and negative reinforcement at the same time are considered the groups at greatest risk of having the worst perception of themselves. Psychologists, educators and therapists who work with this youth population should have some basic knowledge about the heterogeneity of SRB and its relationships with personality variables (e.g., self-concept, anxiety). In addition, they should not forget that during adolescence, rapid changes in terms of physical aspects and constant personal, social and emotional readjustments are experienced by young people [74]. Consequently, in this vulnerable stage of life, to encourage a healthy level of self-concept (e.g., helping youth to set realistic goals, emphasizing each youth’s strengths, facilitating self-praise, establishing a comfortable environments) would contribute to improving their overall sense of well-being [75,76,77].

## Figures and Tables

**Table 1:** Fit indices of the latent class analysis (LCA). The values in bold show the best model fit.

Number of Classes	BIC	Entropy
2 classes	16,572.18	0.69
3 classes	16,208.85	0.68
4 classes	16,034.79	0.69
5 classes	16,024.33	0.65

**Table 2:** Means and standard deviations obtained by the four clusters in Self-Description Questionnaire (SDQ) dimensions.

Dimensions SDQ-II Short ^1^	Moderately High SRB ^2^		Moderately Low SRB ^3^		Mixed SRB ^4^		Non SRB ^5^		Statistical Significance	
	*M*	*SD*	*M*	*SD*	*M*	*SD*	*M*	*SD*	*F_(3,1311)_*	*η^2^*
Physical appearance	11.75	3.92	11.75	3.85	9.86	4.68	12.52	4.18	12.36 *	0.02
Physical abilities	13.92	4.73	13.86	4.54	11.96	5.70	14.33	4.5	7.88 *	0.02
Parent relations	14.61	4.05	15.24	3.91	12.97	4.50	15.64	4.37	14.68 *	0.03
Same-sex relations	17.14	4.21	18.14	3.87	14.49	4.89	18.63	4.26	35.15 *	0.06
Opposite-sex relations	13.02	3.77	13.78	3.62	10.63	4.41	14.48	3.44	34.02 *	0.06
Honesty	18.80	4.77	19.41	4.93	17.60	5.34	20.33	4.92	9.62 *	0.02
Emotional stability	11.42	4.58	13.21	4.51	10.13	4.64	14.11	4.81	35.37 *	0.06
Verbal	13.40	5.56	14.32	5.69	14.17	5.94	13.75	5.41	3.03 *	0.01
Math	10.30	4.96	10.94	5.16	8.55	5.14	10.80	4.93	9.01 *	0.02
General school	12.76	3.98	13.65	3.77	11.60	3.72	13.42	3.99	13.13 *	0.02
Self-esteem	21.54	4.43	22.32	4.32	19.07	5.72	22.74	4.54	22.91 *	0.04

^1^ Self-Description Questionnaire-II Short form; ^2^ Moderately High School Refusal Behavior; ^3^ Moderately Low School Refusal Behavior; ^4^ Mixed School Refusal Behavior; ^5^ Non-School Refusal Behavior *p < 0.001.

**Table 3:** Cohen’s d value for post hoc contrasts between cluster groups on Self-Description Questionnaire (SDQ) dimensions.

Dimensions SDQ-II Short ^1^	Moderately High SRB ^2^ vs. Moderately Low SRB ^3^	Moderately High SRB vs. Mixed SRB ^4^	Moderately High SRB vs. Non SRB ^5^	Moderately Low SRB vs. Mixed SRB	Moderately Low SRB vs. Non SRB	Mixed SRB vs. Non SRB
Physical Appearance	-	0.47	-	0.47	-	−0.60
Physical abilities	-	0.39	-	0.40	-	−0.47
Parent relations	−0.16	0.40	−0.25	0.56	-	−0.60
Same-sex relations	−0.25	0.61	−0.35	0.89	-	−0.91
Opposite-sex relations	−0.20	0.61	−0.39	0.83	-	−0.98
Honesty	-	-	−0.32	0.36	-	−0.53
Emotional stability	−0.39	0.28	−0.58	0.68	-	−0.84
Verbal	−0.16	-	-	-	-	-
Math	-	0.35	-	0.46	-	−0.045
General school	−0.23	0.29	-	0.55	-	−0.47
Self-esteem	−0.18	0.53	−0.30	0.70	-	−0.72

^1^ Self-Description Questionnaire-II Short form; ^2^ Moderately High School Refusal Behavior; ^3^ Moderately Low School Refusal Behavior; ^4^ Mixed School Refusal Behavior; ^5^ Non-School Refusal Behavior.
